# Social capital in a lower socioeconomic palliative care population: a qualitative investigation of individual, community and civic networks and relations

**DOI:** 10.1186/1472-684X-13-30

**Published:** 2014-06-16

**Authors:** Joanne M Lewis, Michelle DiGiacomo, David C Currow, Patricia M Davidson

**Affiliations:** 1 Centre for Cardiovascular and Chronic Care, Faculty of Health, University of Technology Sydney, PO Box 123, Broadway, NSW 2007, Australia; 2 Discipline, Palliative and Supportive Services, Flinders University, 700 Goodwood Rd, Daw Park, South Australia 5041, Australia; 3 School of Nursing, Johns Hopkins University, 525 North Wolfe Street, Baltimore, MD, USA

**Keywords:** Palliative care, End of life care, Lower socioeconomic, Social capital, Networks and relations

## Abstract

**Background:**

Lower socioeconomic populations live and die in contexts that render them vulnerable to poorer health and wellbeing. Contexts of care at the end of life are overwhelmingly determined by the capacity and nature of formal and informal networks and relations to support care. To date, studies exploring the nature of *networks and relations* of support in lower socioeconomic populations at the end of life are absent. This qualitative study sought to identify the nature of individual, community and civic networks and relations that defined the contexts of care for this group.

**Methods:**

Semi-structured qualitative interviews were conducted with 16 patients and 6 informal carers who identified that they had social and economic needs and were from a lower socioeconomic area. A social capital questionnaire identifying individual, community and civic networks and relations formed the interview guide. Interviews were audio-taped, transcribed and analysed using framework analysis.

**Results:**

Participants identified that individual and community *networks and relations* of support were mainly inadequate to meet care needs. Specifically, data revealed: (1) individual (informal caregivers) networks and relations were small and fragile due to the nature of conflict and crisis; (2) community trust and engagement was limited and shifted by illness and caregiving; (3) and formal care services were inconsistent and provided limited practical support. Some transitions in community relations for support were noted. Levels of civic and government engagement and support were overall positive and enabled access to welfare resources.

**Conclusion:**

Networks and relations of support are essential for ensuring quality end of life care is achieved. Lower socioeconomic groups are at a distinct disadvantage where these *networks and relations* are limited, as they lack the resources necessary to augment these gaps. Understanding of the nature of assets and limitations, in *networks and relations* of support, is necessary to inform interventions to improve end of life care for lower socioeconomic populations.

## Background

Socioeconomically disadvantaged populations experience multiple health disparities due to a range of social, economic and exclusion factors which are associated with increased risk for disease and injury and limited use of preventative services [1]. Lower socioeconomic groups are more likely than other socioeconomic groups, to require management of advanced disease [1]; have more complex social and economic needs [2,3]; have more limited awareness and understanding of the principles and practice of palliative care [4]; and were less likely to have a home death [5,6]. These factors indicate different demands and access outcomes for end-of-life care, an often neglected life stage in research on this population [2,3,7,8]. Understanding the experience of end-of-life care for lower socioeconomic groups is necessary, yet perhaps underappreciated amidst the palliative care discourse. End-of-life care consumes significantly the resources of all socioeconomic groups and yet does so disproportionately for individuals and families with the greatest limits of these resources. The nature of *networks and relations* to buffer the drain on resources is understood [9] and important as the health and wellbeing of all individuals and communities is dependent on the nature of their social milieu [10].

### Supportive networks in palliative care

Supportive networks are considered important for emotional support, support for caregiving strategies, information and resource exchange and to enable navigation through a healthcare system [11]. Descriptions and understandings of the range and quality of networks of support in palliative care populations overall is very limited in the literature [12]. The necessity of family caregiving networks to support care for patients is recognised by palliative care philosophy recognising the family as the “unit of care”. The narrow focus on family obligations for caregiving has the potential however to limit wellbeing for the patient and family caregiver [9]. Patients and carers report a desire to have and maintain community and social connections [13].

Formal and informal networks and relationships hold resources for support which have the potential to buffer the effects of social and economic change that precede the diagnosis of a terminal illness. Recognising and optimising networks of support is an emerging priority in healthcare with the aim to engage these networks to potentiate the scarce human and material resources available to patients [14], and more specifically in palliative care populations, to support community engagement for patients and their families at the end of life [15].

The study of social capital has generated understanding of the networks and relations that can create resources [16]. Social capital is understood broadly to be a relational resource; defined as both the individual and collective resources maintained and produced by social relations [16]. The main components of social capital are *Structural* and *Cognitive* (Table 1). The *Structural* social capital component*,* also labeled in the literature as 'network’, considers the formal and informal networks and relations of 'What people do.’ The *Cognitive* social capital component, also termed 'social cohesion’, describes values, perceptions, and norms or the quality of relationships or 'What people feel’ [17]. The distinction between these two components was important as they were seen to have different relationships with health and social outcomes. Harpham [17] described that where there were large networks (structural), of limited quality (cognitive) there was an association with poor mental health outcomes. Social capital more specifically considers both the individual and community resources necessary for buffering the effects of vulnerability and for bridging the structural and cognitive elements of societal conditions. Social capital literature describes the three domains of societal levels to include bonded (individual), bridged (community) and linked (civic) social capital and these are commonly utilised within the discourse of social capital in the literature to describe the interactions of relations within each of the social levels [18] pg 5. The nature and quality of the support provided at these distinct levels therefore matters much to the quality and sustainability of care at the end of life [19].

**Table 1:** Social capital questionnaire domains

**Networks (structural)**	Bonded	Spouse/partner
		Family
		Neighbours
		Friends
**• Formal and informal networks and participations. Distinguished as** ***bonding, bridging*** **and linking networks**	Bridged	Neighbours
		Friends who are not neighbours
		Community/religious groups
		Healthcare providers
**• Objectively observed and measured**	Linked	Government employees, departments
		Politicians
**Cognitive (cohesion/engagement/trust)**	Participation	Family engagement
		Community engagement/volunteering
**• Trust and reciprocity resulting in norms, values and beliefs which support co-operation and engagement in networks.**		Civic engagement
	Support	Emotional
		Economic
		To know things
**• Subjective evaluation.**		To do things
	Trust	Individual (bonded)
		Community (bridged)
		Civic (linked)
	Reciprocity	Willingness to help others

Social capital assessment tools overall aim to report the elements and functions of networks and relations for structure (network) and cognition (cohesion) at different levels of societal connection (individual, community and civic). The evidence for the heterogeneous nature of these networks in caregiving is such that where measured specifically they can be traced for their nuances [9]. Qualitative examination of social capital is limited in the literature and yet is considered a most appropriate method for capturing the contexts and multifaceted nature of the concept [20]. This study utilises qualitative methods to explore the nature of social capital in a socioeconomically disadvantaged group of palliative care patients and carers, using a social capital questionnaire to guide and frame discussions.

## Methods

The study took place in 2011–2012 in a lower socioeconomic area in Western Sydney, Australia, and is located within a larger local government area with a population of just over 300,000 [21]. This is part of a larger study which explored the end of life care needs and capacities of a lower socioeconomic palliative care population. The area in Western Sydney was developed in the mid-1960’s through a public housing program to relocate poor inner city residents to this location on the then city’s boundaries [22]. The Index of Relative Disadvantage (IRSD) of the study recruitment area score is 885 (relative disadvantage associated with scores below 1,000), with low scores representative of disadvantage in income, employment, material and social resources and the capacity to participate in society [23]. IRSD measures socioeconomic status in Australia, from data collected at census and compiled by the Australian Bureau of Statistics.

### Procedures

A purposive sample of participants either self-recruited to the study in response to a recruitment flyer posted in the palliative care facility, or were approached to participate by formal care staff to which they had revealed social and economic hardship needs. The elements of the procedure are described according to the COREQ consolidated criteria for reporting qualitative research [24].

### Data collection

Interviews were semi-structured using an investigator-developed social capital questionnaire (SCQ). The sixteen items comprising the questionnaire were derived from the World Bank’s Social Capital Assessment Tool (SOCAT) [25], the Adapted Social Capital Assessment Tool (ASCAT) [26]; the Household Income and Labour Dynamics Australia (HILDA) survey [27] and the Australian Bureau of Statistics (ABS) General Social Survey [28]. These assessment tools were selected whereby they specifically considered questions for individual, community and organisational elements or were specific questions related to social capital in Australian national surveys. The SCQ developed for the study asked specific questions about networks and relations at individual, community and civic levels. They were also asked about the quality and extent of trust and cohesion within and between these networks and relations. Domains are outlined in Table 1. Five multiple choice questions in the SCQ pertained to descriptions of engagement, support and participation with networks. Participants were asked to elaborate on each of their responses with probes such as 'can you tell me more about that?’ These probes often facilitated lengthy narratives depicting the nature and quality of relations as well as contextual factors, resulting in highly nuanced accounts. The qualitative responses to the SCQ are reported in this paper.

Participants were interviewed by the same female researcher who had worked with the population group for a period of over 10 years. The researcher’s long term relationship with the population enabled access to participants and most importantly engendered an awareness and sensitivity to the stigma of the area and the social and economic challenges for this population. Participants were interviewed in locations according to their preferences; within their own homes or within an in-patient palliative care facility. Participants with full time carers could agree to be interviewed together in a single interview. Interviews lasted an average of 40 minutes and were audio-recorded and transcribed verbatim.

### Ethical consideration

Ethical approval for conduct of the study was obtained from the Western Sydney Local Health District and University of Technology Sydney Human Research Ethics Committees. Written consent was obtained voluntarily from each participant following either self-recruitment or approach to participate in the study. All participants were given pseudonyms during transcription and identifying information was removed from documentation to ensure anonymity.

Researching vulnerable populations requires specific consideration of the risks and harms for the study population. The stigma around defining a population as disadvantaged was addressed carefully in participant information sheets and the recruitment flyer. Patients who had a significant symptom burden or were deteriorating were not recruited. Patients and carers who reported current concerns regarding domestic violence or abuse were also excluded from the study, due the risk for harm related to the questioning around relationship quality.

### Data analysis

Transcripts were analysed using the framework approach [29]. Smith and Firth [30] incorporate Richie and Spencer’s five framework stages into three key steps: data management, descriptive accounts and explanatory accounts. Themes were identified initially with familiarisation of the data and assignment to a category recorded in a coding matrix and partially informed by the instrument. Following coding, data were summarised into initial themes which identified associations and subsequent development of explanatory accounts. To ensure accurate representation, transcripts were checked against recordings by two experienced researchers, which included analysis of field notes. Emergent themes and initial perspectives from the coding matrix were discussed and associations between the themes were considered for patterns and core themes. Divergent cases were included in analysis. Themes were both identified in advance for networks and relations due to the use of a social capital questionnaire and also were derived from the data.

## Results

Sixteen patients and six informal carers agreed to take part in the study. Two participants and carers who were approached to participate in the study declined, one without offering a reason and the other citing concerns for discussing finances, which was an assessment for the larger study. Additionally four participants and/or carers were withdrawn from the study as their condition declined prior interview and two others were withdrawn (with their approval) as they revealed concerns for answering questions about close relationships due to issues of domestic violence.

Table 2 outlines the participant characteristics. The median range for patient age was 61–70 years and participants were predominantly men. Only six of the patients in the study had full-time, live-in family carers while another eight patients had family caregivers who provided intermittent care. Two patients reported no caregiving support from family and described neighbours as providing intermittent caregiver support. One patient who was married described no caregiving support from his spouse.

**Table 2:** Socio demographic characteristics of study participants

**Socio-demographic characteristics**			
		**Patients (n = 16)**	**Carer (n = 6)**
Mean age		66.3	56.8
Age	˂31	1	0
	31-40	0	1
	41-50	1	1
	51-60	4	2
	61-70	3	0
	71-80	5	2
	81-90	2	0
Gender	Male	9	2
	Female	7	4
Diagnosis	Malignant	14	N/A
	Non-malignant	2	N/A
Marital status	Married/partner	7	6
	Widowed	4	0
	Single	5	0

### Bonded care conditions-small and fragile

Bonded care conditions summarised the state and capacity of family and other close neighbour relations of support for end of life care. These relationships enabled care at home for patients. Overall bonded care conditions were described as small in terms of the numbers of family members available to provide support and fragile in terms of the quality of these relations.

### Fragile relations

Patient participants were open in disclosing relationship concerns in the interviews. A patient, Mary, laughed when given the option to describe the quality of the relationship with her spouse: '*It’s not excellent…fair’*. David, a patient, described very limited support from his partner and said *'I am not even sure why I am even with her*.’ The discussion of family relations for several patients heralded descriptions of larger contexts of alcoholism and violence that had shaped the quality and nature of family relations that continued during their end of life care period. Ruth, a patient who lived alone with support from her son and daughter, was fearful that their visits might reveal her whereabouts to her ex-husband, who had been violent with her throughout their marriage and break-up.

'Great lengths have been taken and he doesn’t know where I live’.

Ruth declined to spend her last weeks of life at home or to be cared for by her children in their homes. A lone end-of-life was preferable in her circumstances.

Carers in the study also reported relationship concerns regarding other family members contributing to caregiving. The limits of support from other family members and pre-existing relationship difficulties with family members were highlighted by several caregivers. One daughter caring for her father described how her siblings were not forthcoming with caregiving support, stating that support was *'not very much,’* and was only provided when care was '*really needed’*. Other caregivers in the study discussed concerns for limited support from family somewhat obscurely in interviews; highlighting that it was an issue but not appearing to want to discuss their concerns further in front of their loved one. One male caregiver reported that when he became frustrated and overwhelmed when caring for his wife he would go out in the garden and take his frustrations out with a pick axe, to dig the ground and *'curse the daughter’*. Overall, provision of informal care was by a sole caregiver with some intermittent support from other family members.

### Bonds of care

Two patients who lived alone confirmed established neighbour networks which were invaluable for sustaining many care and social needs. One of these patients had family living interstate and the other had been estranged from his wife and children for many years. These patient participants both lived in government (welfare) housing complexes and described the support from neighbours increasing as they required it. One 83 year old gentleman, Harold, described many of his neighbours as great support which was both practical and emotional. He described discussing, at length, the outcomes of doctor’s appointments with neighbours, often from which these neighbours had transported him.

Steven, a patient who also lived alone without family support, had a very close relationship with his neighbour, Peter, and described Peter’s extended support during his stay in the palliative care facility:

'He bought me pyjamas, of course, paid the light bill, paid the phone bill and he pays my rent, so he does a good job.’

Steven’s neighbour was nominated by him to become his Power of Attorney and he additionally included him in decisions regarding his healthcare choices.

Some patients in the study described new relations with neighbours since diagnosis of their terminal illness. They reported that neighbours mowed their lawns and delivered meals. Two patients, who had recently relocated to the area from rural areas, reported no relationships with neighbours and '*absolutely no*’ support from them.

### Bridged care conditions-limitations, loss and transition

*Bridged care conditions* reflect accounts for the nature of the community context of cohesion and trust, social engagement and formal care support. Communities were overall limited in their provision of support (formal and informal) and social engagement was sometimes lost in illness and caregiving, and other times transitioned to supportive contexts for some participants.

### Area cohesion

The semi-structured interview responses to the SCQ elicited discussion from participants about their reasons for 'migration’ to the area. Patients and carers described the social and economic context of the area and levels of cohesion in the area and overall described their community as their local community, their neighbourhood area. Many participants described moving to the area as adults with young families or with their parents as dependents. Three participants relocated to the area following their own diagnosis of a terminal diagnosis or that of a loved one, to be closer to family and/or to access cheaper housing. These participants highlighted the stigma attached to the area as one with a large lower socioeconomic demographic. Participants who had lived in the area for many years did not discuss concerns over the socioeconomic demographic, but overall did not perceive levels of community trust or cohesion.

Mary, a patient who had lived in the area for several decades, qualified that her community held up the appearance of being cohesive. In responding to the question '*do most people in the community get along well?’* Mary highlighted that her community '*appeared to get along well’* however most residents did not necessarily engage with one another.

'I’d like to believe that, but they really don’t communicate. When I look around, there are no real problems in my street around me, but I hardly know anyone either, so I’d say, yeah, they generally get on well, mainly because they just don’t communicate with one another.’

Paul, a patient who lived in a larger complex of government housing units, reiterated this experience of community disconnect. He explained that his relationship with neighbours was *'alright’* because of their limited contact.

'Neighbours-Oh they’re there alright. They leave me alone and I leave them alone.’

Paul went on to describe his concerns for increasing refugee migration to the area and increasing concerns for safety in his neighbourhood. The limits of communication with other cultural groups and the increase in numbers in culturally diverse populations in the neighbourhood were concerns raised by Paul and two other participants and led to more limited community engagement for several.

### Social loss

Some patients described a loss of social contact due to illness and disability and this was more pronounced in patients who lived alone. Winston, an 83 year old gentleman, described his inability to continue with regular visits to the local Returned Services League (RSL) club to meet friends and enjoy social engagement, because he had been diagnosed with *'this bloody malarkey’*. Winston also described valuable social connections within his local area, which were no longer possible due to his illness.

'Before I got stuck with this bloody cancer, we’d go across early in the morning and chat with the newsagent and some of the early people and we’d discuss everything that was going on.

Family caregivers also described a loss of social engagement One carer reported feeling 'homebound’ and that outside of home no longer represented a place that they felt they belonged. John, a 53 year old carer, described it in this way:

'Because it’s not the norm anymore. It’s the exception, rather than the normality of it. You feel uncomfortable outside of that cocoon, I guess. If you need to go out, you feel uncomfortable’

Mary, a patient with head and neck cancer, found social engagement difficult. She felt very self-conscious about social outings because of her facial disfigurement and thus, avoided these when alone.

'Well, we used to go down the club. I don’t go on my own as I feel very self-conscious.’

Patients and carers described a range of social networks and clubs and many expressed the desire to maintain these connections. Many patients and carers were still attending their local sports or recreation club and although they described limited interaction with other patrons, it was a location for them to meet up, be entertained, and be a part of a 'social world’.

### Formal care margins

Patients and carers discussed formalised community care support as overall being somewhat inconsistent and unpredictable. Formal healthcare services were mainly provided by community nurses and general practitioners (GPs). Two carers were receiving personal care for their spouses with longer term care needs from a non-government provider through a specific funded government program. These community care networks were supported in the community by some adjuvant specialist palliative care nursing and medical services.

When asked about the level of support provided by community nursing services many patients and carers were not sure of the formal care being provided by these services, as they were unsure how to describe the support in practical terms. Mark, a patient whose main carer was his elderly mother, commented on the limited nature of this practical support describing only weekly visits and reporting that the support provided by the nurse was *'just advice’* . Other reports of limited contact with formal nursing services were described, with one patient, Winston, describing his community nursing service as ad-hoc, stating that the service did not operate *'on any timetable’*. Another patient reported that he was unsure if he was in receipt of a service.

'I don’t know. The nurse I’ve got now I’ve never even met her because they keep changing them’. (Patient, aged 53)

Although descriptions of inconsistencies in formal service provision were confirmed by several participants, it was the limited nature of practical hands-on support from community nursing services described most consistently across the participant group, which highlighted a greater concern.

### Linked conditions-trust and resources

Linked conditions of macro-level engagement enabled access mainly to financial benefits and housing resources which were important for participants in the study who were receiving government welfare payments or reported loss of income due to illness and caregiving requirements. The relationships with inter-sectoral agencies were important for sustaining or procuring access to many of the material resources required for daily living costs and caring needs and costs for this group.

### Contacts for resources

All participants in the study described contact with government agencies which mainly consisted of welfare support agencies and government housing departments. Participants who described receiving government support benefits such as the aged or disability pension for periods which preceded their illness or caregiving periods described that their engagement with these agencies was generally positive. When additional or advance payments were needed, participants reported receiving these without too many problems. Participants identified that direct face-to-face contact with staff in welfare agencies was overall easily achievable and made a difference to the outcome. One patient, David, discussed how he had had a most positive engagement with the Government Housing Department and that they were most efficient and prompt in finding public housing for him at short notice.

Alternatively, participants who were new to receiving government benefits described their negotiations with these agencies as being more difficult. These participants described being unfamiliar with the processes involved in applying for and maintaining benefits. One carer, Marion, described 'giving up’ on an application for financial benefits because of the complexity of the process and not having the 'emotional energy’ to continue to engage with agencies that were unsympathetic and uncaring. The quality of the interaction with government agencies and their providers was important for access to material resources for this group.

Descriptions of engagement with members of local or state government describe the broadest relations to positions of authority and additionally provided further links to material resources. When participants were asked about having contact recently with politicians or Member of Parliament, several laughed and denied having any such contact. Nearly a quarter of participants in the study did, however, report contact. Some participants reported that they had acquaintance with one local government member or state politician, others however reported that they were acquainted with several politicians and that the relationship with these person(s) was positive and they considered there to be potential for future support.

'I know quite a few as friends [politicians]. If I needed them, they’d be there, I know that.’ (Patient, aged 55)

'I know the Mayor of Blacktown and I know the Mayor of Penrith and yeah, I’m sure they’d help if I asked them, but I haven’t had to.’ (Carer, aged 75)

Access to resources was an important outcome of the engagement with politicians and participants who had such contact described both this and the ease of access to such persons. David, a patient who needed urgent public housing, contacted his local Member of Parliament without any difficulties and received a response and support in a timely manner.

## Discussion

The SCQ uniquely assessed the nature of social context for a lower socioeconomic population of patients at the end of life and their family carers. The social capital approach to understanding needs and capacities of lower socioeconomic populations is important for understanding the contribution of *networks and relations* to buffer the levels of disadvantage in health populations [31]. Understanding the relations and networks of support for lower socioeconomic populations with end of life care needs is invaluable for supporting the increased needs of this group and ascertaining a rich description of their social milieu. A more sustainable and considered health promotion approach to palliative care, which shifts attention away from health service development to consider the resources which likely engender community engagement and can ensure greater sustainability of resources for patients and carers at the end of life is needed [32].

The limits of informal (bonded) care relations for quality and quantity described by patients and carers in this study are supported by the discourse in the literature on the nature of bonded networks for disadvantaged populations. Existing research reports that informal family networks and relations in disadvantaged populations can be inadequate in their capacity to support caregiving, due to the nature of family conflict [7] and additionally due to the limits of resources available to this group [33,34]. The model of informal family caregiving, revered in palliative care theory, therefore requires a rethink most urgently, particularly in socioeconomically disadvantaged populations [7]. The gaps in caregiving *networks and relations* for this study population highlight the risks for poor end of life care outcomes unless additional and/or alternative relations or networks of support are identified and resourced.

Where informal care resources are limited or unavailable, other capacities of community (bonded) networks for supporting and sustaining caregiving and community engagement are necessary. Limited social networks and lack of social support [35], limited neighbourhood contexts for services and facilities [10] and poor community trust outcomes [36] describe the contexts of communities of disadvantage. Participants in this study overall reported low levels of community trust and engagement, which likely would have impacted further on levels of social isolation and limited family networks. The nature of migration to the area due to its cheaper housing, the limits of resources available in such a disadvantaged area and concerns for community engagement due to increasing cultural diversity, all potentially contributed to the limited community engagement and cohesion in this population. The significance of community context is therefore highlighted as an important measure of caregiving capacity and wellbeing in disadvantaged palliative care populations and should be considered in needs assessment, policy and research.

Formal palliative care services were noted in the literature for being inadequate to meet end of life care needs [37-40]. Additionally, informal caregivers of palliative care patients were reported to be managing large caregiving responsibilities and workloads for assessment, symptom management, personal care and household duties [41-44]. Not surprisingly, unmet needs and caregiver burden were reported in this study. Most alarming, however, are these outcomes in a population that lacks the human and material resources to augment the gaps between formal and informal care contexts. Descriptions in the literature for disparity in access to palliative care services for lower socioeconomic groups highlight further disadvantage for this group [6,45].

Some descriptions of capacity in *networks and relations* were described by patients and carers in the study. Civic or government (linked) networks and relations, important for enabling access to social and financial resources, were overall described positively by interview participants for gaining necessary resources and services. These outcomes contradict the literature which reports that lower socioeconomic groups were less likely to engage at the macro level as they tended to have lower levels of trust for positions of government and authority [46]. The distinction of positive civic engagement and trust in this group may represent the norms of trust developed at this level due to positive outcomes of financial support through welfare payments [47]. Additionally such engagement can represent a trend for social activism which in disadvantaged populations can arise from the management of social exclusion at other societal levels [10,22] or as an outcome of political agency due to compulsory electoral engagement (voting) [48]. The drivers for civic engagement are likely complex in this population, the nature of trust and engagement at this broadest societal level, however, denotes a capacity for social cohesion and access to resources which can engender wellbeing in this population.

The attention to quality and quantity of relations is important to ensure relations and networks are developed across societal levels to ensure access to resources and alleviate social exclusion [31]. These aims are important and likely increasingly important, for meeting the end of life care needs of populations and those most at risk for poor outcomes due to limited networks and relations. The social capital framework enabled description and evaluation of the caregiving *networks and relations* which underpinned the context of end of life care and caregiving for this population. Understanding determinants that can impede or enable caregiving and wellbeing is important and can support targeted interventions to better manage end of life care.

### Limitations

This study had a number of limitations, in particular that persons who described circumstances of domestic violence were withdrawn from or not recruited to the study due to safety concerns. This group represent a most vulnerable and excluded group for whom contexts were not well described in this study; that is with the exception of some discussion from participants who were no longer in relationships with their violent partners and described some impacts for these circumstances. Another limitation is that the heterogeneity of relations and networks are such that capturing this complexity in an interview structured for responses to a 16 item questionnaire was difficult. Some patients and carers in the study found it difficult to summarise, for example, the range and quality of relations with siblings as these often differed significantly. The qualitative methodology of the study enabled participants to somewhat articulate this heterogeneity in interviews, but likely they did not describe a complete picture of their relationship contexts. Despite this, descriptions of context, relations and community were rich for describing experience and meaning for these patients and carers.

## Conclusion

This article documented the nature of individual, community and civic *networks and relations* of a lower socioeconomic population at the end of life. The limited capacity of individual (informal caregivers) networks and relations and low levels of community trust and engagement are such that the context of end of life care for this group is likely inadequate for achieving quality outcomes and sustaining home care. The transition of some neighbour networks to provide support for this group and levels of civic trust and engagement reported by participants, highlight some capacities in community and civic networks as assets for this population. Further research for the nature of social capital in disadvantaged populations can further define needs and assets of care contexts for this population.

## Competing interests

The authors declare that they have no competing interests.

## Authors’ contributions

JML, MD and PML designed the study and conducted data analysis. JML conducted data collection and wrote the first draft of the manuscript. All authors contributed to the critical revision and approved the final content.

## Pre-publication history

The pre-publication history for this paper can be accessed here:

http://www.biomedcentral.com/1472-684X/13/30/prepub
